# Phylogeography of the Olive Ridley Sea Turtle (*Lepidochelys olivacea*): Standardized Global Haplotype Nomenclature and Updated Mitochondrial Lineage Distributions in the Eastern Pacific

**DOI:** 10.3390/ani16193021

**Published:** 2026-09-25

**Authors:** Omar Horacio Sol-Torres, Hugo David Gallardo-Sánchez, Fátima Yedith Camacho-Sánchez, Sarah Maria Vargas, Eduardo Resendiz, Alan A. Zavala-Norzagaray, José Alberto Narváez-Zapata, Luis A. Tello-Sahagún, Catherine E. Hart, José Alfredo González-Gerardo, Raymundo Rosas-Quijano, Didiana Gálvez-López, Miguel Angel Reyes-López

**Affiliations:** 1 Laboratory of Conservation Medicine, Centro de Biotecnología Genómica, Instituto Politécnico Nacional, Reynosa 88700, Tamaulipas, Mexico; osolt2400@alumno.ipn.mx (O.H.S.-T.); hgallardos2400@alumno.ipn.mx (H.D.G.-S.); 2 UAM Reynosa-Aztlan, Universidad Autónoma de Tamaulipas, Reynosa 88740, Tamaulipas, Mexico; facamacho@uat.edu.mx; 3 Laboratório de Genética e Evolução Molecular, Universidade Federal do Espírito Santo, Vitória 29075-910, Brazil; sarah.vargas@ufes.br; 4 Departamento Académico de Ciencias Marinas y Costeras, Universidad Autónoma de Baja California Sur (UABCS), La Paz 23080, Baja California Sur, Mexico; jresendiz@uabcs.mx; 5 Instituto Politécnico Nacional (IPN-CIIDIR Sinaloa), Guasave 81049, Sinaloa, Mexico; anorzaga@ipn.mx (A.A.Z.-N.); lats.255@gmail.com (L.A.T.-S.); cehart03@gmail.com (C.E.H.); 6 Laboratorio de Biotecnología Industrial, Centro de Biotecnología Genómica, Instituto Politécnico Nacional, Reynosa 88740, Tamaulipas, Mexico; jnarvaez@ipn.mx; 7 Estación Biológica Majahuas, Playón de Mismaloya, Tomatlán 48400, Jalisco, Mexico; 8 Grupo Tortuguero de las Californias A.C., La Paz 23090, Baja California Sur, Mexico; 9 Uella Environmental Research and Consulting, Bahía de Banderas 63732, Nayarit, Mexico; 10 Centro de Promoción Ambiental, Museo Regional Comunitario de la Tortuga Marina, Ejido Emiliano Zapata, Mazatán 30655, Chiapas, Mexico; biogongerja2@gmail.com; 11 Instituto de Biociencias, Universidad Autónoma de Chiapas, Tapachula 30798, Chiapas, Mexico; raymundo.rosas@unach.mx (R.R.-Q.); didiana.galvez@unach.mx (D.G.-L.)

**Keywords:** *Lepidochelys olivacea*, phylogeography, nomenclature, haplotypes, East Pacific, Mexico, genetic diversity

## Abstract

*Lepidochelys olivacea* is the most abundant sea turtle species worldwide and exhibits a circumtropical distribution across the Atlantic, Pacific, and Indian Oceans. Global phylogeographic studies have identified three major mitochondrial lineages, but comparisons among regions remain limited by inconsistent mtDNA control-region fragment lengths, incompatible haplotype nomenclatures, and uneven geographic sampling. The East Pacific Regional Management Unit (RMU), which includes major nesting populations in Mexico and Costa Rica, experienced severe demographic declines during the twentieth century, and the effects of expanded sampling on current phylogeographic patterns remain uncertain. This study analyzed 1264 mtDNA control region sequences (474-bp and 653-bp fragments) to update and standardize the global genetic dataset of *L. olivacea*, incorporating novel samples from previously unsampled East Pacific localities. Clear phylogeographic differentiation among RMUs was detected and remained consistent across both sequence lengths, supporting the Atlantic (ATL), East Pacific (EP), and Indo-West Pacific (IWP) lineages. Shared haplotypes between the EP and IWP lineages likely reflect historical connectivity rather than contemporary transoceanic movements. The 653-bp dataset revealed greater haplotype diversity (78 haplotypes) than the 474-bp dataset (62 haplotypes), highlighting the improved resolution provided by the longer marker. Regional diversity patterns appeared to be influenced by sampling effort, particularly in underrepresented regions such as the West Indian Ocean. Negative neutrality indices in both Pacific RMUs suggested recent population expansion. This study establishes a standardized haplotype nomenclature for *L. olivacea*, provides an updated view of mitochondrial lineage distributions, and offers a robust framework for future phylogeographic and conservation studies across the species’ range.

## Simple Summary

The olive ridley sea turtle (*Lepidochelys olivacea*) is the most abundant sea turtle species worldwide, yet many populations remain vulnerable because of historical overexploitation and ongoing human activities. Effective conservation depends on understanding how populations are related and how genetic diversity is distributed throughout the species’ range. However, previous genetic studies have been difficult to compare because they analyzed different portions of the same genetic marker and used inconsistent names for the same genetic variants, limiting global assessments of population structure and connectivity. This study updated and standardized the global genetic database for olive ridley turtles by integrating previously published information with new samples from previously unsampled nesting beaches in the East Pacific. The analyses confirmed three major genetic groups distributed across the Atlantic, East Pacific, and Indo-West Pacific regions and showed that longer DNA sequences reveal greater genetic diversity than shorter ones. A standardized naming system for genetic variants was also established, allowing future studies to be directly compared. This work provides an updated framework for understanding the evolutionary history and geographic distribution of genetic diversity in olive ridley sea turtles and offers a valuable resource for future research, long-term population monitoring, and conservation planning throughout the species’ global distribution.

## 1. Introduction

*Lepidochelys olivacea* (Eschscholtz, 1829) is the most abundant sea turtle species worldwide, exhibiting a circumtropical distribution that spans the Atlantic, Pacific, and Indian Oceans across more than 80 countries [1]. Through its foraging activities, nesting behavior, and nutrient transport, this species contributes substantially to the functioning of both coastal and pelagic marine ecosystems [1,2]. Despite its global abundance, the olive ridley is currently classified as vulnerable by the IUCN [3] due to an estimated population decline of approximately 30% worldwide [1]. Consequently, understanding patterns of connectivity and population structure across its distribution is essential for developing effective conservation and management strategies.

The mitochondrial DNA (mtDNA) control region, or D-loop, has been the primary genetic marker used to investigate phylogeographic patterns in sea turtles. This marker is widely employed due to its relatively high mutation rate, which provides sufficient variation to resolve population structure, its largely neutral evolution, and its maternal inheritance, which allows tracking of female-mediated dispersal [4]. However, maternal inheritance also represents a limitation, as it captures only the female component of gene flow and does not undergo recombination, which can constrain inferences about certain demographic processes depending on the biological question being addressed. These studies have provided the basis for delimiting Regional Management Units (RMUs), which represent demographically independent populations that serve as the principal framework for sea turtle conservation planning [5]. In *L. olivacea*, phylogeographic analyses suggest that oceanographic barriers and historical dispersal events have shaped contemporary patterns of genetic diversity and connectivity among ocean basins [4,6,7].

Comparability among published studies is further hindered by the absence of standardized nomenclature frameworks not only in *L. olivacea* but also in other sea turtle species and marine vertebrates, where independent haplotype designations and variable fragment lengths complicate cross-study integration. Variation in mtDNA fragment lengths and the independent designation of haplotypes across studies have generated incompatible nomenclatures, complicating comparisons among regions and through time. As a result, lineage distributions and patterns of genetic diversity may be incompletely characterized, reducing the reproducibility and comparability of phylogeographic inferences. Given the continued accumulation of genetic data, the development of a transparent and standardized nomenclature framework has become increasingly important for global-scale analyses of *L. olivacea*.

These challenges are particularly relevant in the East Pacific RMU, which includes some of the largest nesting populations of the species, particularly in Mexico and Costa Rica. During the second half of the twentieth century, intensive commercial exploitation caused severe demographic declines throughout the region, resulting in the collapse of several major nesting colonies [8,9]. Although subsequent conservation measures have promoted population recovery, genetic studies continue to report low levels of population differentiation across East Pacific rookeries [4,10]. Nevertheless, differences in haplotype composition among nesting sites and the incorporation of newly available samples suggest that regional diversity may remain incompletely documented, warranting a reassessment of current phylogeographic patterns.

Given these limitations, the present study aimed to update and standardize the global mtDNA control region dataset of *L. olivacea* to reassess the phylogeographic distribution of mitochondrial lineages across the species’ range. To achieve this, we integrated newly available sequences from public repositories together with novel samples from previously unsampled East Pacific localities, established a standardized haplotype nomenclature for both short and long mtDNA fragments, and evaluated patterns of genetic diversity, differentiation, and connectivity within a global phylogeographic framework.

## 2. Materials and Methods

### 2.1. Study Site

The study was conducted along the Mexican Pacific coast, on both nesting and foraging grounds previously and newly sampled, located in the states of Chiapas, Jalisco, and Sinaloa (Figure 1). Previously sampled sites included Emiliano Zapata Beach (EZB); Boca de Tomates (BTC); and Las Glorias Beach (LGB), while newly sampled sites were Majahuas Camp (MC) and Navachiste Bay (NB). These localities were selected to represent different aggregation types across the species’ regional distribution range.

The Mexican Pacific coast encompasses a diversity of nesting habitats, ranging from solitary nesting beaches to mass-nesting arribada sites, and includes important foraging grounds influenced by the California Current, the North Equatorial Current, and seasonal upwelling events. These oceanographic features contribute to high primary productivity and support critical habitats for olive ridley turtles across different life stages. The Pacific Ocean is the most volcanically active oceanic province due to the interaction of the Pacific and North American plates [11,12], which has facilitated the formation of unique geological formations supporting diverse marine ecosystems [13]. Coastal habitats across the study area include mangroves, estuaries, lagoons, dunes, and rocky reefs, which support diverse marine communities and important nesting habitats for multiple sea turtle species [12].

### 2.2. Sample Collection

A total of 37 was obtained from the Latin American Sea Turtle Biobank at the Conservation Medicine Laboratory from Centro de Biotecnología Genómica, Instituto Politécnico Nacional. Samples were collected between 2017 and 2025 under permits issued by the DGVS-SEMARNAT (N° SGPA/DVGS/003769/18; N° SGPA/DVGS/00998/22; and N° SGPA/DVGS/06414/23).

Blood samples were collected from nesting females during oviposition, whereas tissue samples were obtained from free-ranging juveniles captured in offshore waters and from unborn hatchlings. Sampling included Playa Las Glorias (n = 5 nesting females; n = 2 unborn hatchlings), Bahía de Navachiste (n = 7 juveniles), Campamento Tortuguero Majahuas (n = 3 nesting females), Campamento Tortuguero Boca de Tomates (n = 3 nesting females), and Playa Emiliano Zapata (n = 17 nesting females). Venipuncture was performed on the jugular vein using sterile 20 G needles, and blood was stored in lithium-heparin Vacutainer tubes (Becton Dickinson, Franklin Lakes, NJ, USA) under cold chain conditions at −80 °C. Conversely, muscle tissue samples were obtained from non-emerged hatchlings and small (3 mm) biopsy samples from juvenile flippers. Tissue fragments were preserved in 70% ethanol and stored at 4 °C until processing.

### 2.3. mtDNA Extraction and Amplification

Genomic DNA was extracted from blood and muscle using the Qiagen DNeasy Blood&Tissue extraction kit (Qiagen, Valencia, CA, USA), following manufacturer protocols. DNA quality and concentration were assessed using a NanoDrop spectrophotometer (Thermo Fisher Scientific, Waltham, MA, USA) and 1% agarose gels.

The mtDNA control region was amplified following the protocol of Vilaça et al. [4] using primers LCM15382 (forward: 5′-GCTTAACCCTAAAGCATTGG-3′) and H950 (reverse: 5′-GTCTCGGATTTAGGGGTTTG-3′) [14]. PCR reactions were performed in 25 μL of master mix containing 1× PCR buffer, 1.5 mM MgCl_2_, 0.2 mM each dNTP, 0.4 μM each primer, and 1 U of Taq DNA polymerase. The reaction conditions consisted of an initial denaturation at 95 °C for 3 min, followed by 35 cycles of 94 °C for 1 min, 53 °C for 1 min, and 72 °C for 1 min, with a final extension at 72 °C for 10 min. PCR products were evaluated using 2% agarose gels.

### 2.4. Sequencing and Processing

PCR amplicons were purified using the ethanol precipitation method. Purified products were resuspended in nuclease-free water, quantified, and sent for sequencing by Eurofins Genomics (Louisville, KY, USA) using Sanger sequencing technology.

In parallel, a global reference database of mtDNA control region sequences and complete mitochondrial genomes of *L. olivacea* was compiled from NCBI GenBank (accessed until August 2025) and from the dataset of Vilaça et al. [4]. All sequences are identified by their GenBank accession numbers, which are provided in Supplementary Table S3. The original source publication for each sequence can be traced through its corresponding GenBank record and, where applicable, through the supplementary data of Vilaça et al. [4]. The NCBI database samples were used to complement and update the original haplotype and frequency sequence dataset reported by Vilaça et al. [4]. Sequences were filtered using a Q40/Len ≥ 0.80 quality criterion. Low-quality sequences were manually trimmed or excluded in BioEdit v7.2.5 [15].

All sequences were aligned using ClustalW v2.1 implemented in R v4.3.0 [16]. To evaluate the effect of sequence length on estimates of genetic diversity and population structure, while maximizing comparability with previously published reference sequences, two independent datasets were generated. The first dataset was trimmed to 653 bp to match the sequence length reported by Vilaça et al. [4]. The second dataset was trimmed based on the AF051776.1 reference sequence from Bowen et al. [6], resulting in a final alignment length of 474 bp.

### 2.5. Haplotype Nomenclature

Due to substantial variation in sequence lengths and inconsistencies in haplotype nomenclature among published studies of *L. olivacea*, a standardized global haplotype nomenclature was established following the criteria of the Archie Carr Center for Sea Turtle Research. Haplotypes identified in the Atlantic RMUs were designated as LoA-X, whereas those from the Indian and Pacific oceans were designated as LoIP-X to account for the high number of shared haplotypes between these regions. It is important to note that the LoA and LoIP designations represent operational groupings intended to facilitate global haplotype standardization and long-term genetic monitoring of the species, rather than a definitive statement of fixed geographic origin. Haplotype distributions are not static and may change over time through dispersal and demographic processes; therefore, this nomenclature should be viewed as a pragmatic framework for standardization purposes. Within each designation, numerical identifiers were assigned according to haplotype frequency, beginning with the most abundant haplotypes and continuing in descending order of occurrence.

To ensure full reproducibility and transparency of the nomenclature system, the following criteria were applied: (1) Prioritization of existing names: When multiple names referred to the same sequence, we prioritized the most frequently used name in the literature, followed by the earliest published name, cross-referencing with the original GenBank accession to confirm identity; (2) Sequence orientation and fragment coordinates: All sequences were aligned to the AF051776.1 and AY920519.1 reference sequences [6], with orientation standardized to the forward strand using the LCM15382 primer as the start reference, and trimmed to 474 bp and 653 bp, respectively; (3) Treatment of gaps, ambiguous bases, and indels: Gaps were treated as missing data, sequences with ambiguous bases (N, R, Y, etc.) were included only when ambiguous positions did not affect haplotype assignment, and indels were treated as single mutational events following the TCS algorithm; (4) Reconciliation of identical sequences with different published names: We manually curated all sequences with conflicting names by comparing underlying sequences and cross-referencing with GenBank accessions, retaining the most commonly used name and listing all alternatives in Supplementary File S1 as “Previous Assignment”; (5) Numbering of previously unnamed haplotypes: Previously unnamed haplotypes were numbered sequentially (LoAX or LoIPX) in descending order of frequency, with new haplotypes detected only in the 653 bp fragment designated as subhaplotypes (e.g., LoA2.1, LoA2.2); (6) Submission to a recognized haplotype registry: We have not yet submitted the proposed nomenclature to a formal registry, as no single international registry exists for sea turtle mitochondrial haplotypes, but we have deposited the full nomenclature table (Supplementary File S1) with the manuscript and will make it publicly available through Supplementary Materials and, if possible, through a public repository such as FigShare or Dryad, and we intend to share the nomenclature with the Archie Carr Center for Sea Turtle Research and the IUCN Marine Turtle Specialist Group for broader community adoption; and (7) Addition of future haplotypes: To prevent future naming conflicts, we propose that new haplotypes be added by continuing the numerical sequence (e.g., LoA10, LoIP66) and, where applicable, as new subhaplotypes (e.g., LoA2.4) when they differ only in the extended 653 bp region.

Haplotype frequencies were estimated by pooling nesting and oceanic aggregations within each RMU to establish a standardized nomenclature representing the complete haplotype diversity currently documented for each RMU. Genetic diversity and population structure analyses were performed separately for nesting and oceanic aggregations when appropriate.

Because shorter mtDNA fragments may collapse multiple long-fragment haplotypes into a single sequence variant, the 474 bp dataset was used to define additional haplotypes within the 653 bp sequences. Specifically, haplotypes that differed in the 653 bp alignment but became identical after truncation to 474 bp were grouped under the same short-fragment haplotype designation.

### 2.6. Genetic Diversity and Population Differentiation

Population structure and genetic diversity analyses were performed independently for nesting and oceanic aggregations, considering each RMU as a population. Genetic diversity was assessed by estimating the number of polymorphic sites (S), number of haplotypes (H), nucleotide diversity (π), and haplotype diversity (Hd) using Arlequin v3.5 [17]. Population structure was evaluated for nesting aggregations using AMOVA based on pairwise *F_ST_* estimates in Arlequin v3.5 with 10,000 permutations. To further evaluate genetic differentiation within the East Pacific RMU, additional comparisons were performed by including Mexico and Costa Rica separately in the global analyses.

Additionally, following the phylogeographic framework of Vilaça et al. [4], pairwise *F_ST_* comparisons among the Atlantic (ATL), East Pacific (EP), and Indo-West Pacific (IWP) lineages were performed using nesting aggregations grouped by lineage.

### 2.7. Haplotype Network and Demographic Analyses

Haplotype relationships were reconstructed using the Templeton, Crandall, and Sing (TCS) algorithm in networks implemented in PopART [18]. Haplotypes were grouped according to frequency and geographic distribution. Both the dominant haplotype and general spatial patterns were visualized for each RMU using QGIS v3.34 [19].

Historical demographic patterns and recent colonization events were evaluated using Tajima’s D [20] and Fu’s Fs [21] analyses in Arlequin v3.5. Negative values of Tajima’s D and Fu’s Fs were interpreted as signatures of recent population expansion, whereas positive values were considered indicative of balancing selection or demographic stability.

To further investigate demographic history, mismatch distribution analyses were performed for the East Pacific and West Pacific RMUs using Arlequin v3.5. The sum of squared deviations (SSD) and raggedness index (Hri) were calculated to test the fit to a sudden expansion model, with statistical significance assessed through 10,000 bootstrap replicates. Multimodal mismatch distributions were interpreted as indicative of demographic stability or complex historical fluctuations, while unimodal distributions were considered consistent with recent population expansion.

## 3. Results

A total of 1264 sequences were compiled from NCBI GenBank and Vilaça et al. [4]. Each sequence is identified by its GenBank accession number in Supplementary Table S3, which allows the original source of each sequence to be traced. The East Pacific and West Pacific RMUs were the most represented among *L. olivacea* studies, comprising 52.85% of all reported sequences (Figure 2). In contrast, the West Indian RMU was the least represented, accounting for only 2.69% of the total sequences. Additionally, as reported for other sea turtle species, a strong sampling bias toward nesting site studies was observed, with nesting populations representing 82.43% of all compiled sequences.

### 3.1. Genetic Diversity

The longer-fragment dataset allowed the identification of 78 haplotypes, whereas the short-fragment dataset yielded 62 haplotypes. The East and West Pacific RMUs exhibited the highest haplotype diversity, while the West Indian RMU showed the lowest diversity (Table 1 and Table 2). As observed between well-represented and underrepresented regions, sampling bias may substantially influence estimates of genetic diversity. Similar patterns were observed in the number of polymorphic sites and nucleotide diversity.

### 3.2. Haplotype Nomenclature

Within the 474 bp alignment, nine haplotypes were identified for the Atlantic group, designated as LoA1–LoA9. In the 653 bp alignment, three additional haplotypes were detected within LoA2 (LoA2.1–LoA2.3), while two additional haplotypes were identified for both LoA3 and LoA6.

For the Indo-Pacific group, 53 haplotypes (LoIP1–LoIP53) were identified in the 474 bp sequences, whereas the 653 bp alignment resolved 65 haplotypes, allowing the recognition of 12 additional haplotypes.

Haplotype nomenclature and pooled haplotype frequencies for each RMU, including both nesting and oceanic aggregations, are provided for both datasets in Supplementary Tables S1 and S2. Complete sequence metadata, including GenBank accession numbers, sampling locality, RMU, aggregation type (nesting or oceanic), and sequence, are provided in Supplementary Table S3. The alignment files with standardized haplotype names are provided as Supplementary Table S4.

To facilitate the adoption of the proposed nomenclature by other researchers and to enable seamless integration with previously published data, we have prepared a comprehensive correspondence table (Supplementary File S1). This table lists all previously published haplotype names alongside their corresponding new designations for both 474 bp and 653 bp fragment lengths. For each haplotype, the table includes the Haplowebmaker ID, geographic region (Atlantic or Indo-Pacific), representative GenBank accession number, and total frequency observed in this study. Additional haplotypes resolved exclusively in the longer fragment are indicated with “.1”, “.2”, etc., allowing researchers working with different fragment lengths to identify the equivalent haplotype in either dataset. This correspondence table serves as a bridge between historical nomenclature and the standardized framework proposed here, greatly facilitating cross-study comparisons and the long-term utility of the genetic data generated for this species.

All haplotypes detected in both newly and previously sampled areas in this study corresponded to previously reported haplotypes, herein designated as LoIP1, LoIP4, LoIP12, LoIP17, LoIP24, and LoIP27 in the 474 bp alignment, and as LoIP1.1, LoIP4.1, LoIP12.1, LoIP17.1, LoIP24.1, and LoIP27.1 in the 653 bp alignment.

The geographic distribution of haplotypes revealed distinct dominant maternal lineages among RMUs (Figure 3). Specifically, the East Pacific RMU was characterized by the predominance of LoIP1.1, whereas LoIP2.1 was the most frequent haplotype in both West Pacific and Northeast Indian RMUs. Conversely, the most frequent haplotype for West Indian RMUs was LoIP10.1, while both Atlantic (East and West) RMUs showed a marked dominance of the haplotype LoA1.1.

Although RMUs represent genetically coherent management units, regional differences in haplotype composition can occur within their distributional range. Consistent with this pattern, 25 previously reported haplotypes were detected only in Mexico using the 653 bp length (LoIP1.1, LoIP1.2, LoIP4.1, LoIP5.1, LoIP8.1, LoIP8.2, LoIP9.1, LoIP11, LoIP14.1, LoIP17.1, LoIP18.1, LoIP19.1, LoIP19.2, LoIP24.1, LoIP26.1, LoIP27.1, LoIP28.1, LoIP31.1, LoIP32.1, LoIP38.1, LoIP46.1, LoIP47.1, LoIP48.1, LoIP49.1, and LoIP52.1), despite Mexico and Costa Rica both belonging to the East Pacific RMU.

### 3.3. Genetic Connectivity and Demography

Pairwise *F_ST_* analyses among nesting sites revealed substantial variation in genetic differentiation across populations. Using the 474 bp dataset (Figure 4A), *F_ST_* values ranged from 0.023 to 0.836, with 31 significant pairwise comparisons. The highest differentiation was observed between WA and WI (*p* < 0.05), whereas the lowest values occurred between EA and WA (*p* < 0.05). Notably, Mexico and Costa Rica exhibited very low and non-significant genetic differentiation (*F_ST_* = 0.015).

The analysis based on the 653 bp dataset (Figure 4B) recovered a similar pattern of genetic structure, with pairwise *F_ST_* values ranging from 0.051 to 0.801. Significant differentiation was detected in 35 comparisons. The longer fragment thus produced more significant pairwise comparisons (35 vs. 31) and a 25% increase in haplotype diversity (78 vs. 62 haplotypes), demonstrating improved statistical power and resolution.

Across both marker lengths, the general pattern of genetic structure was consistent, although the 653 bp dataset provided greater resolution for detecting significant population differentiation.

Pairwise *F_ST_* comparisons between nesting aggregation lineages revealed that the ATL exhibited high and significant genetic differentiation from the EP (*F_ST_* = 0.792, *p* < 0.05), whereas differentiation between ATL and IWP populations was lower but remained significant (*F_ST_* = 0.386, *p* < 0.05). Comparisons between the East Pacific and Indo-West Pacific groups revealed moderate genetic differentiation (*F_ST_* = 0.41, *p* < 0.05).

Across both datasets, significant Tajima’s D and Fu’s Fs values in nesting populations were detected only in the EP and WP RMUs. All significant estimates were negative, whereas Fu’s Fs for the WP RMU in the short-fragment dataset was negative but not significant (Table 1).

### 3.4. Mismatch Distribution and Demographic Analyses

To further investigate the demographic signals indicated by Tajima’s D and Fu’s Fs, mismatch distribution analyses were conducted for the East Pacific and West Pacific RMUs using only the 653 bp sequences, which exhibited stronger and more statistically significant values in Tajima’s D analyses. The East Pacific RMU exhibited a unimodal mismatch distribution (SSD = 0.008, *p* = 0.452; Hri = 0.034, *p* = 0.521), consistent with a model of recent population expansion. In contrast, the West Pacific RMU displayed a multimodal mismatch distribution (SSD = 0.021, *p* = 0.231; Hri = 0.056, *p* = 0.187). The mismatch distribution results thus provide independent support for the demographic interpretations derived from the neutrality tests.

The haplotype networks revealed several haplotypes shared among Indo-Pacific RMUs, whereas multiple haplotypes were exclusive to the Atlantic region. Three major genetic clusters were consistently recovered in both datasets: two clusters comprising mixed Indo-Pacific RMUs and a third cluster grouping with individuals almost exclusive to the Western and Eastern Atlantic (WA and EA). Consistent with the pairwise *F_ST_* analyses, haplotype networks revealed the greatest genetic divergence in the EP lineage, which was separated from both ATL and IWP lineages by a larger number of mutational steps.

Despite recovering the same major genetic groups, the long-fragment dataset provided greater haplotype resolution, revealing additional variants and mutational connections that were not detected in the short-fragment dataset.

## 4. Discussion

Here we propose the first global nomenclature standardization for *L. olivacea* haplotypes using two different sequence lengths. Although several studies have developed independent haplotype nomenclatures or relied on nomenclatures that are not publicly available, differences in sequence lengths and fragment positions among studies resulted in overlapping codenames and potential misidentification [4,6]. In such an instance, we developed a new haplotype nomenclature while evaluating genetic diversity, differentiation, and connectivity of previously published and newly generated sequences from throughout the species’ range.

### 4.1. Phylogeographic Patterns

Our results reveal evident phylogeographic differentiation for *L. olivacea* RMUs worldwide, a pattern that remained consistent across both sequence lengths. Notably, each RMU was characterized by a dominant haplotype, with LoIP1.1 in the East Pacific; LoIP2.1 in the West Pacific and Northeast Indian; LoIP10.1 in the West Indian; and LoA1.1 in both the East and West Atlantic. This pattern is consistent with the recognized physiographic barriers to dispersal and long-term gene flow, which promote divergence between populations from different ocean basins [4,23]. While this framework agrees with the study of Vilaça et al. [4], the comparison of short and long sequences allowed greater genetic resolution for the identification of additional haplotypes and significant *F_ST_* comparisons. The high pairwise *F_ST_* values (Figure 4) and the clustering patterns observed in the haplotype networks (Figure 5 and Figure 6) support the delimitation of Atlantic, East Pacific, and Indo-West Pacific RMUs as distinct phylogeographic lineages, as previously described by Vilaça et al. [4].

Given the geographic proximity and the presence of several shared haplotypes between Indo-West Pacific and East Pacific populations, lower differentiation between these Pacific lineages than between Atlantic and Indo-West Pacific lineages might be expected. However, haplotype networks showed that East Pacific haplotypes were separated from the other lineages by a greater number of mutational steps, suggesting that shared haplotypes may reflect historical connectivity while the East Pacific lineage has followed a distinct evolutionary trajectory. In contrast, the Atlantic lineage did not share haplotypes with either Indo-West Pacific or East Pacific populations yet exhibited a similar level of differentiation (*F_ST_*) from the Indo-West Pacific lineage. This pattern is consistent with the phylogeographic scenario proposed by Vilaça et al. [4], in which Atlantic populations represent a recent recolonization derived from Indo-West Pacific ancestors, as supported by the close relationship between Atlantic and Indo-West Pacific clades and the occurrence of an Indo-West Pacific-derived central haplotype within the Atlantic lineage. Conversely, East Pacific and Atlantic lineages are not directly related and are separated by a high *F_ST_* value, suggesting that contemporary patterns of differentiation reflect distinct recolonization histories from ancestral Indo-Pacific populations rather than geographic distance alone.

Although other studies have interpreted evidence from both mtDNA and microsatellite markers, including inferred migrants, admixed individuals, and estimates of gene flow between RMUs, as support for transoceanic connectivity, these signals should be interpreted within a broader ecological and evolutionary context [4,10]. Unlike loggerhead and green turtles, in which transoceanic movements have been directly documented through satellite telemetry, no study has yet demonstrated migrations between East Pacific and Indo-West Pacific populations or between Indo-West Pacific and Atlantic populations of *L. olivacea*. Furthermore, olive ridleys exhibit ecological and physiological characteristics generally associated with more restricted migratory behavior and a greater dependence on neritic habitats than other sea turtle species [24,25]. This is consistent with studies suggesting that olive ridley females adopt an income breeding strategy, foraging in the vicinity of nesting grounds during the reproductive season to sustain annual and repeated clutch production without incurring the energetic costs of long-distance migrations [26]. Consequently, the genetic affinities observed among these regions may not necessarily reflect ongoing transoceanic dispersal. Instead, they may represent the genetic signature of historical recolonization events from ancestral Indo-West Pacific populations, as previously proposed by Vilaça et al. [4]. Under this scenario, patterns interpreted as evidence of gene flow may instead reflect the retention of ancestral polymorphisms and historical dispersal events rather than frequent contemporary migration across ocean basins.

Among all evaluated RMUs, only East Pacific and West Pacific populations exhibited consistently significant negative Tajima’s D and Fu’s Fs values. The mismatch distribution analyses provided independent support for these demographic interpretations. The East Pacific displayed a unimodal mismatch distribution, consistent with recent population expansion following a bottleneck, which aligns with the historical record of intensive commercial exploitation in the region during the twentieth century followed by subsequent recovery [8,9,27]. In contrast, the West Pacific exhibited a multimodal mismatch distribution, suggesting a more complex demographic history with sustained population expansion over a longer evolutionary timescale, consistent with the proposed Indo-West Pacific origin of *L. olivacea* and the persistence of large source populations in this region [4,6,7]. Such patterns are expected when populations recover from demographic reductions, generating an excess of rare haplotypes relative to neutral expectations [20,21,28]. The combined evidence from neutrality tests and mismatch distributions thus supports the interpretation of recent expansion in the East Pacific and longer-term demographic stability with gradual expansion in the West Pacific.

### 4.2. Research Gaps in Genetic Monitoring

In contrast to genetic differentiation, genetic diversity estimates were moderately influenced by fragment length, with longer sequences recovering a slightly greater number of haplotypes. However, regional patterns of diversity appeared to be driven not only by marker resolution, but also by sampling effort. The highest levels of haplotype diversity were consistently observed in the most intensively studied regions (more sequences), suggesting that current estimates may partially reflect uneven geographic sampling. Consequently, the low diversity detected in underrepresented regions such as the West Indian should be interpreted cautiously, particularly considering that the Indian Ocean supports some of the largest nesting populations of olive ridley, with three arribada beaches in India accounting for more than 100,000 nests per year [1,7]. Additional sampling in these areas may therefore reveal a greater proportion of the species’ genetic diversity than is currently recognized.

Despite the absence of population structure among nesting sites within the East Pacific RMU, differences in haplotype composition were observed between Mexico and Costa Rica, with several haplotypes detected exclusively in Mexico. This pattern may reflect local variation in haplotype diversity; however, it could also be influenced by differences in sampling effort and sequence availability among regions. As additional rookeries are sampled and new datasets become available, some of these apparently exclusive haplotypes may be detected elsewhere within the East Pacific, providing a more complete characterization of regional genetic diversity.

Because the monitoring of oceanic aggregations is technically challenging and economically demanding, genetic information remains extremely limited. This represents a major knowledge gap, as oceanic aggregations provide access to males and non-nesting life stages that are largely absent from the majority of studies. Consequently, estimates of genetic diversity and differentiation derived from oceanic aggregations are strongly influenced by small sample sizes and uneven sampling effort. Several RMUs included fewer than five individuals, limiting the statistical power of neutrality and differentiation analyses and potentially obscuring biologically meaningful patterns. Therefore, the low levels of genetic diversity and the reduced number of significant pairwise comparisons observed in some oceanic aggregations should be interpreted cautiously, as they may primarily reflect sampling limitations rather than the underlying genetic characteristics of these aggregations.

A further challenge in the compilation and analysis of public genetic data is the potential for sample duplication, where the same individual may have been included in multiple studies without the authors’ awareness. This issue is particularly relevant for widely studied species such as *L. olivacea*, where tissue samples or DNA extracts may be shared among research groups, or where identical sequences from the same location and year may inadvertently be counted multiple times. To address this limitation, future studies should consider implementing standardized individual identification systems, for example using microsatellite- or SNP-based genetic barcoding, to enable unambiguous tracking of individuals across datasets and prevent unintended duplication. Such approaches would greatly enhance the reliability and reproducibility of meta-analyses and comparative phylogeographic studies.

### 4.3. Genetic Standardization

Comparisons among published studies of *L. olivacea* are often complicated by differences in mtDNA fragment length and haplotype nomenclature. As a result, integrating datasets from different regions or time periods may require additional sequence trimming and nomenclatural reconciliation, potentially limiting direct comparisons among studies.

To improve dataset compatibility, we implemented a standardized haplotype nomenclature and evaluated diversity patterns using both short and extended mtDNA fragments. Although both marker lengths recovered similar phylogeographic patterns, longer fragments provided greater haplotype resolution while remaining compatible with historical datasets.

The correspondence table provided in Supplementary File S1 represents a key step toward resolving nomenclature inconsistencies, as it allows researchers to easily translate previously published haplotype names into the standardized framework proposed here. This will facilitate the integration of historical data with new findings and promote the adoption of a unified nomenclature across the field. We have also included a recommendation (Section 2.5) that future haplotypes be added by continuing the numerical sequence (e.g., LoA10, LoIP66) and, where applicable, as new subhaplotypes (e.g., LoA2.4) when they differ only in the extended 653 bp region, and that researchers consult Supplementary File S1 before assigning new names to avoid duplication.

Future genetic surveys would benefit from clearly reporting sequence lengths, trimming criteria, and haplotype designation methods, as well as making updated haplotype reference tables publicly available. Such practices would facilitate comparisons among studies, improve the integration of newly generated data, and contribute to more consistent long-term genetic monitoring of *L. olivacea*.

### 4.4. Conservation Implications

From a conservation perspective, genetic diversity alone may not be sufficient to identify priority populations. While some regions exhibited limited genetic characterization, lower diversity, or unique haplotypic composition, conservation value should also consider factors such as population size, demographic trends, threat exposure, degree of protection, and the availability of genetic information. Nevertheless, populations from the Indian Ocean that remain poorly studied or underrepresented in genetic datasets warrant particular attention, as their contribution to the species’ overall genetic diversity remains largely unknown [4]. Likewise, the Indo-West Pacific lineage harbors some of the highest levels of genetic diversity reported for the species and has been proposed as a central component of the historical processes that shaped the contemporary distribution of *L. olivacea*, highlighting its importance for preserving the species’ evolutionary potential [4,6,7]. Finally, populations or nesting sites characterized by private or regionally restricted haplotypes may contribute unique components of genetic diversity and should be considered in conservation planning to maintain evolutionary distinctiveness across the species’ range.

## 5. Conclusions

This study provides the first global standardization of *L. olivacea* haplotype nomenclature across two sequence lengths, establishing a unified classification framework to ensure consistency and comparability across future genetic investigations of the species. The stability of the observed patterns across diversity, differentiation, and connectivity estimates corroborates the robustness and utility of this framework as a reliable foundation for future research. Moreover, the global synthesis of existing *L. olivacea* sequences reveals critical sampling gaps, underscoring the need for targeted genetic studies in underrepresented regions such as the Indian Ocean. Genetic characterization of these regions is especially critical given the role of the Indian Ocean, as it constitutes the ancestral origin of the species’ worldwide distribution. Additionally, this study expands the existing genetic dataset of olive ridley in the East Pacific RMU by incorporating samples from individuals across different life stages, obtained from both previously studied and newly surveyed nesting sites and foraging grounds. Nonetheless, oceanic aggregations remain critically underrepresented in the current global genetic dataset, despite harboring individuals from multiple populations and life stages that are essential for a comprehensive understanding of connectivity and population dynamics. In this context, we propose that future research should: (1) prioritize closing both geographic and aggregation-type gaps through targeted field sampling in underrepresented regions; (2) deposit available genetic data in open-access repositories to enhance data utility; and (3) adopt a single publicly available haplotype nomenclature to maximize the comparability and long-term utility of mitochondrial data in genetic monitoring.

## Figures and Tables

**Figure 1:**
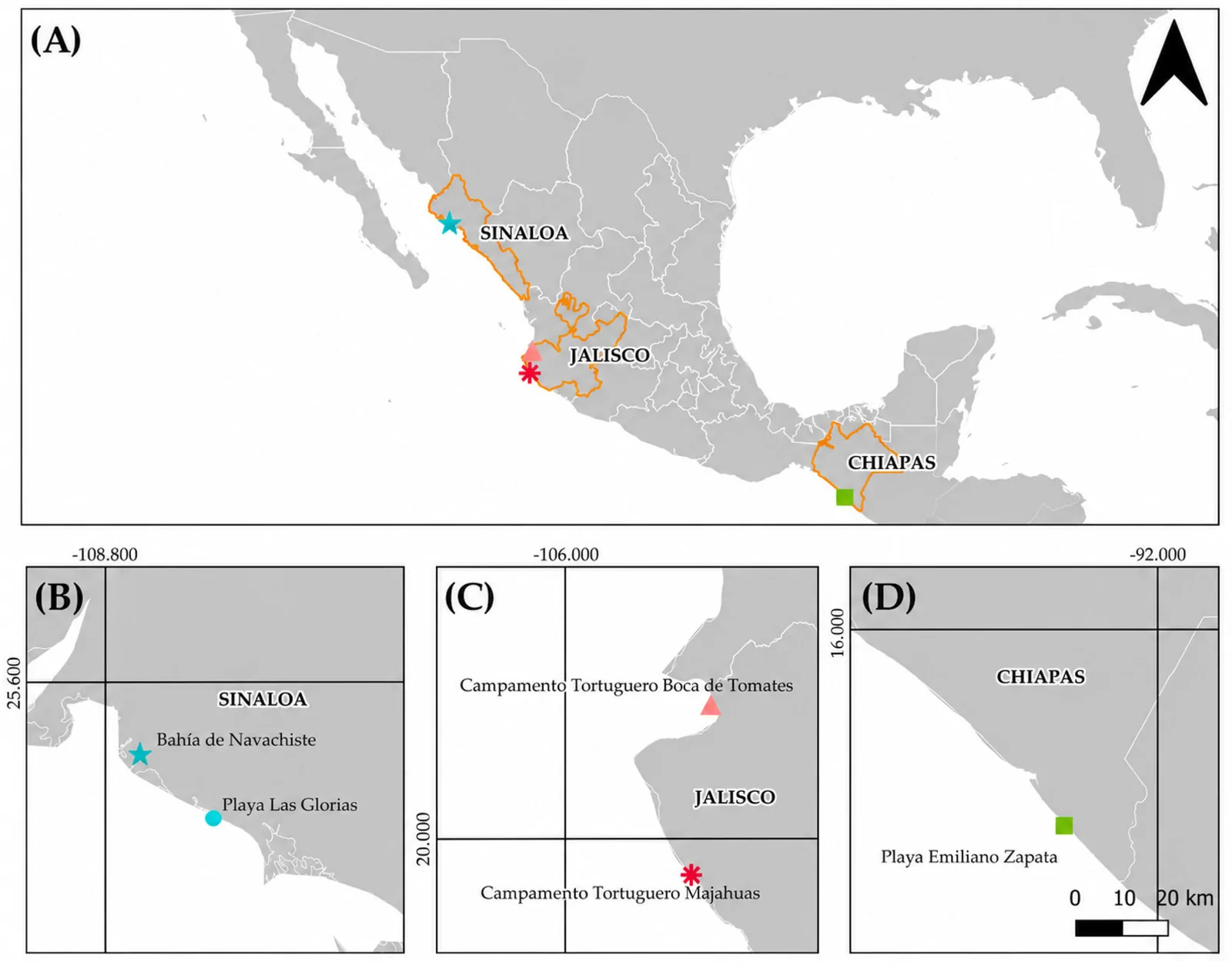
Sampling sites of *Lepidochelys olivacea* along the Mexican Pacific coast: (**A**) overview map showing the location of the states of Sinaloa, Jalisco, and Chiapas; (**B**) detailed view of Sinaloa showing Bahía de Navachiste (star) and Playa Las Glorias (dot); (**C**) detailed view of Jalisco showing Campamento Tortuguero Boca de Tomates (triangle) and Campamento Tortuguero Majahuas (asterisk); and (**D**) detailed view of Chiapas showing Playa Emiliano Zapata (square). Grid lines represent geographic coordinates, and the scale bar is shown in kilometers.

**Figure 2:**
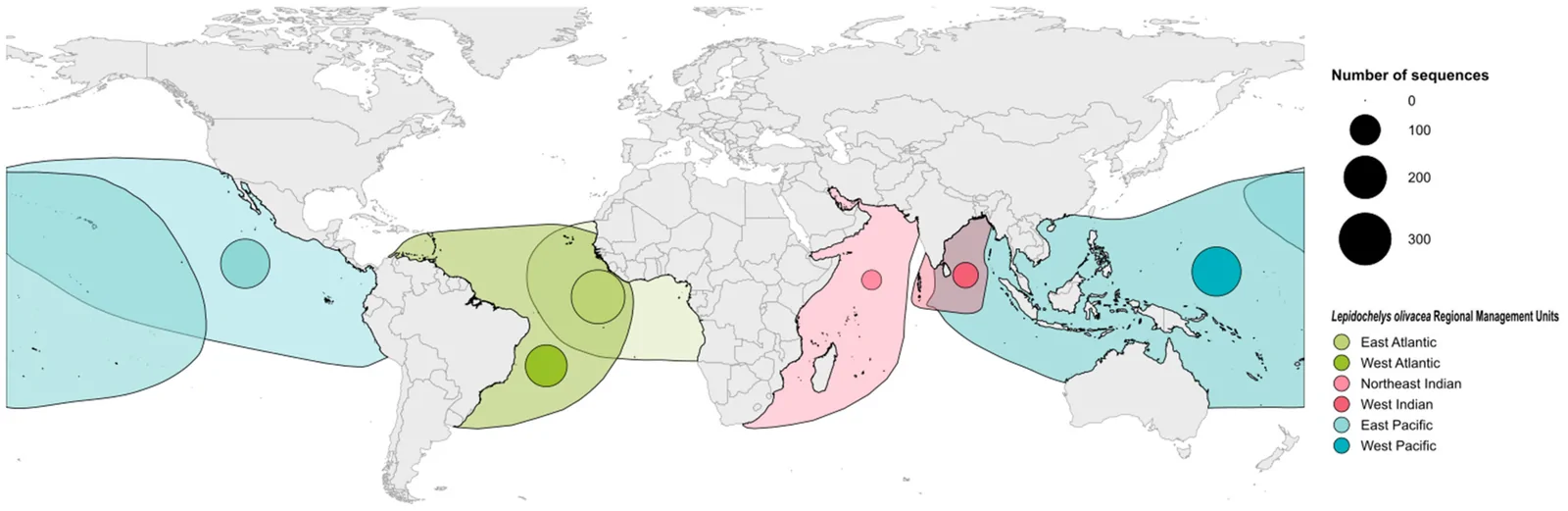
Global distribution of mtDNA control region sequences of *Lepidochelys olivacea* compiled in this study, showing the number of sequences recovered per country. Color intensity reflects the relative number of sequences per country, with darker shades indicating higher sampling effort.

**Figure 3:**
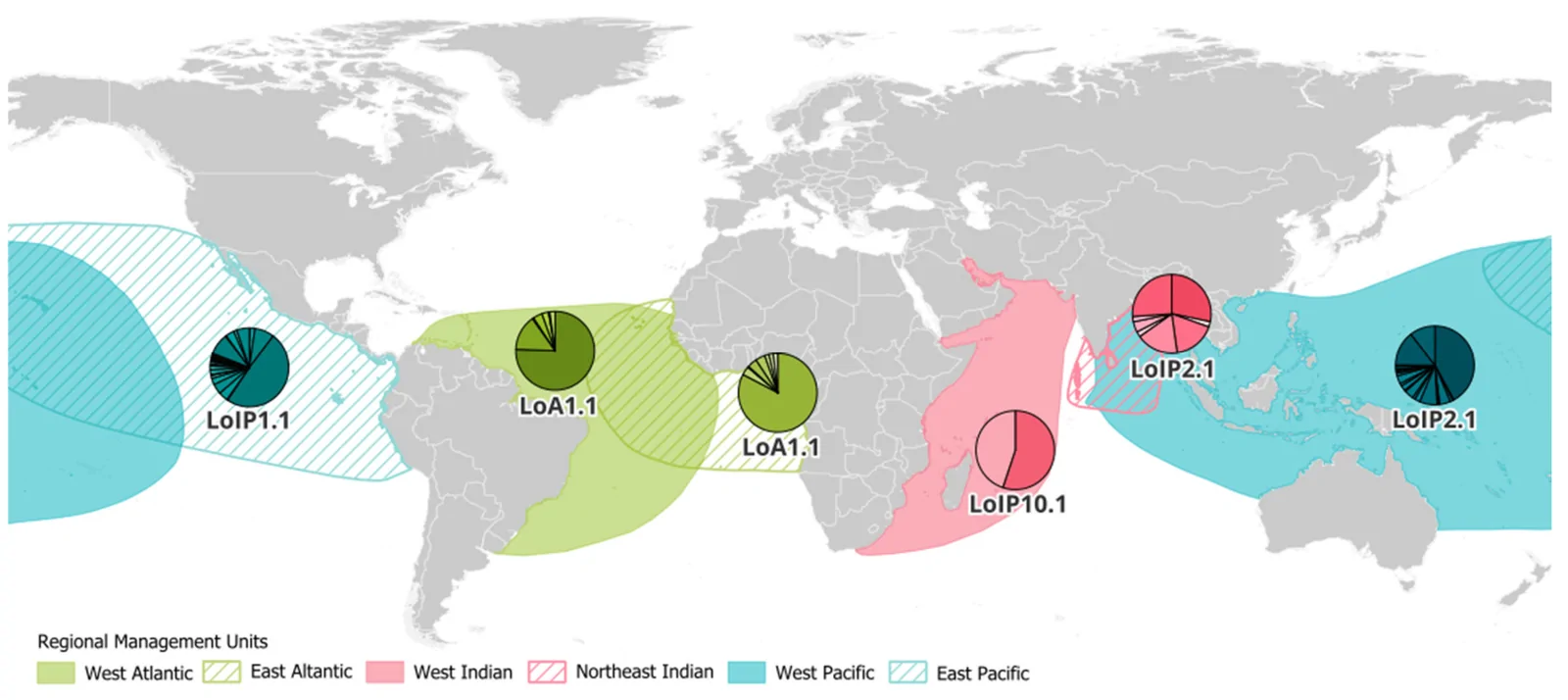
Dominant haplotypes (assigned) and less frequent haplotypes (unassigned) of *Lepidochelys olivacea* across Regional Management Units (RMUs), according to Wallace et al. [5]. Assigned haplotypes refer to the dominant haplotype within each RMU that has been given a specific name/code (e.g., LoA1.1, LoIP1.1), while unassigned haplotypes include all other haplotypes combined. Each RMU is represented by a distinct color, and the pie charts within each RMU indicate the relative frequency of the dominant haplotype (solid color) versus all other haplotypes combined (shaded areas). It is important to note that haplotypes are shared across RMUs, particularly within the Indo-Pacific region, and the figure illustrates relative frequencies rather than haplotype exclusivity. The shapefile for RMU delimitation was obtained from the SWOT database [22].

**Figure 4:**
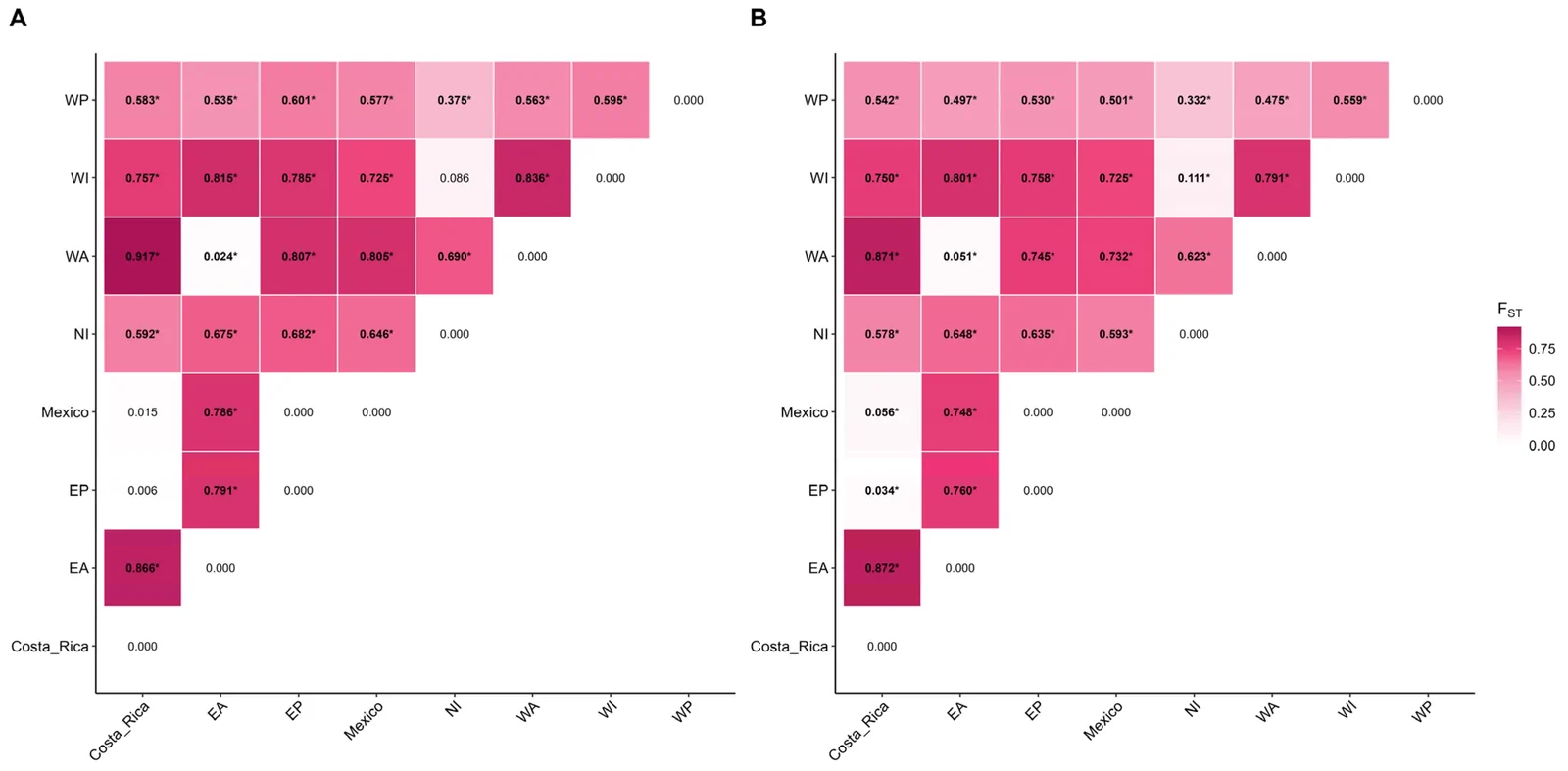
Pairwise *F_ST_* estimations of *Lepidochelys olivacea* nesting populations across Regional Management Units (RMUs). Heatmap color intensity represents the degree of genetic differentiation, with darker colors indicating greater population divergence. (**A**) Analyses based on the 474 bp dataset. (**B**) Analyses based on the 653 bp dataset. Asterisks (*) indicate statistical significance (*p* < 0.05). Abbreviations: EP = East Pacific; WP = West Pacific; EA = East Atlantic; WA = West Atlantic; NI = Northeast Indian; WI = West Indian.

**Figure 5:**
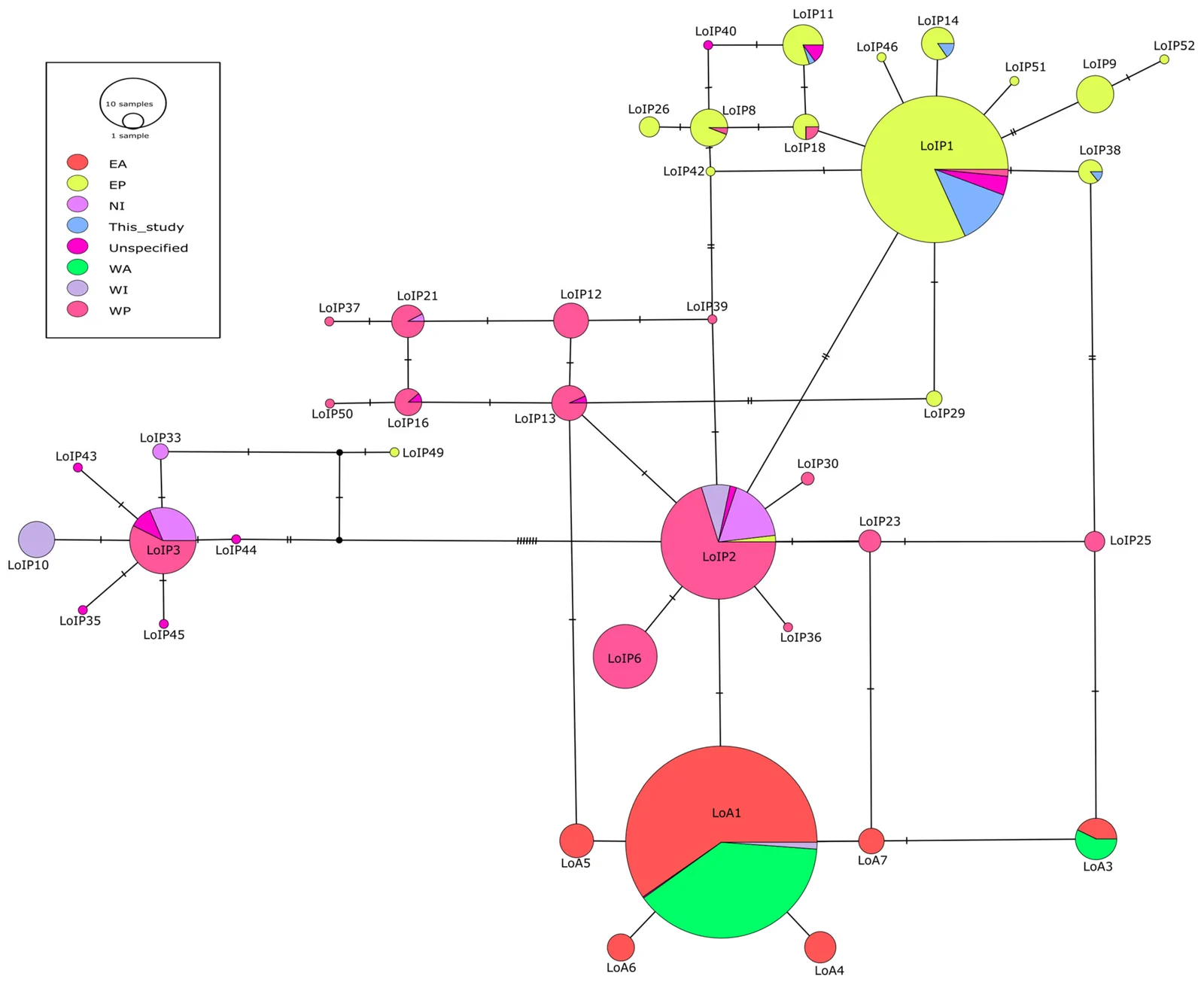
Haplotype network of *Lepidochelys olivacea* based on the 474 bp mtDNA control region fragment. Each node represents a distinct haplotype, with node size proportional to haplotype frequency. Colors indicate the Regional Management Unit (RMU) in which each haplotype was detected. Small black bars along the branches represent mutational steps between haplotypes. Haplotype codes follow the standardized nomenclature proposed in this study (LoA for Atlantic haplotypes; LoIP for Indo-Pacific haplotypes).

**Figure 6:**
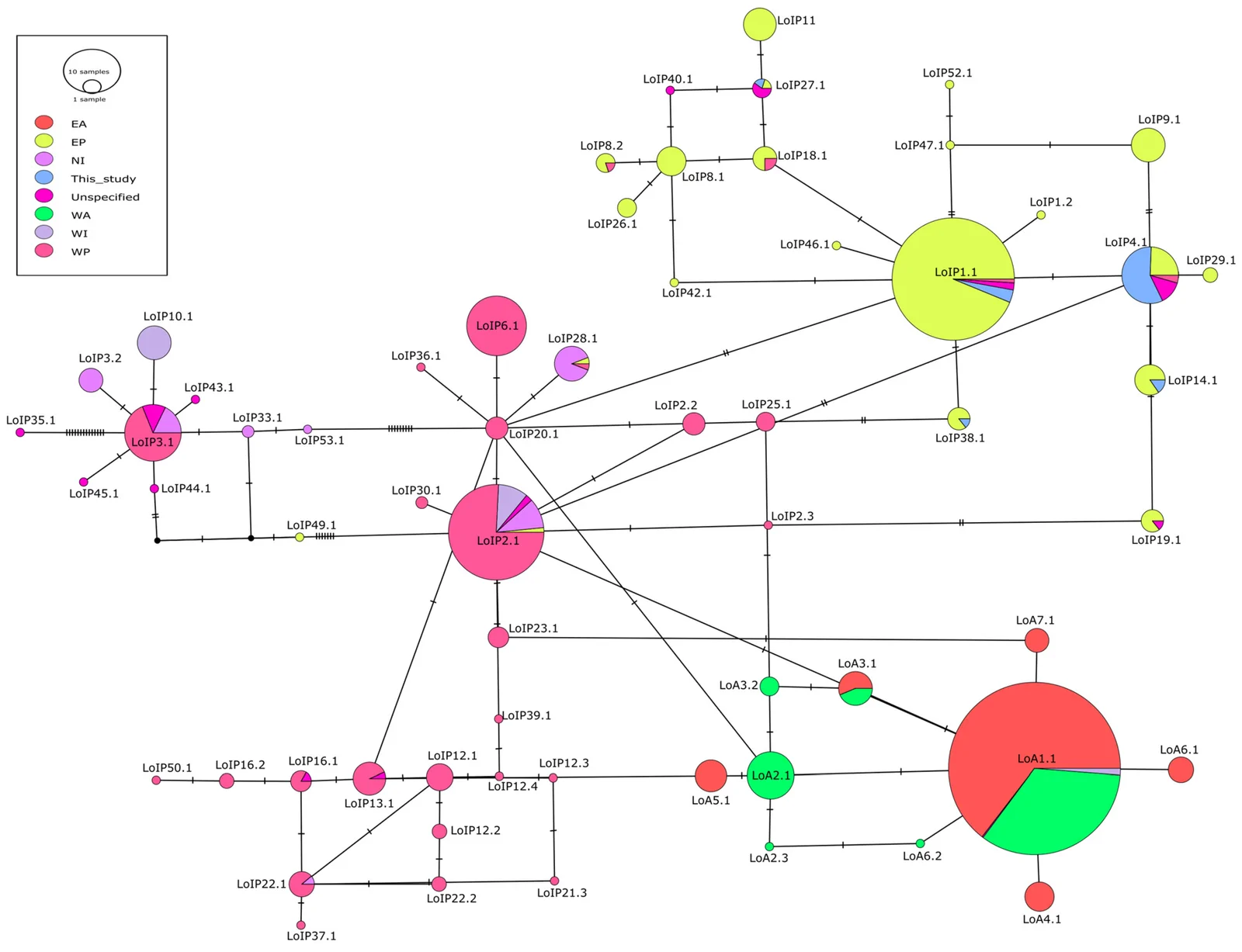
Haplotype network of *Lepidochelys olivacea* based on the 653 bp mtDNA control region fragment. Each node represents a distinct haplotype, with node size proportional to haplotype frequency. Colors indicate the Regional Management Unit (RMU) in which each haplotype was detected. Small black bars along the branches represent mutational steps between haplotypes. Haplotype codes follow the standardized nomenclature proposed in this study (LoA for Atlantic haplotypes; LoIP for Indo-Pacific haplotypes). The longer fragment allowed the resolution of additional haplotypes (indicated by “.1”, “.2”, etc.) that were collapsed in the shorter fragment analysis. The network was constructed using the TCS algorithm implemented in PopART.

**Table 1:** Genetic diversity indices of *Lepidochelys olivacea* nesting populations across RMUs.

Dataset	Statistics	MEX	CR	EP	WP	EA	WA	NI	WI
474 bp	n	231	75	310	181	225	185	48	29
	*S*	21	8	27	24	6	1	11	11
	*H*	24	10	29	20	8	4	7	2
	*Hd*	0.8057	0.5268	0.7536	0.7522	0.3126	0.4002	0.7394	0.5123
	*π*	0.0025	0.0013	0.0022	0.0038	0.0008	0.0002	0.0111	0.0118
	*Tajima’s D*	−1.7239 *	−1.5238 *	−2.0060 *	−1.5671 *	−1.1956	−0.3003	3.3414	3.281
	*Fu’s Fs*	−4.3349	−4.2512 *	−9.2507 *	−4.7566	−3.8733	0.0043	9.8152	13.4952
653 bp	n	231	73	308	181	225	184	46	29
	*S*	25	6	27	29	7	3	14	11
	*H*	27	9	31	23	8	7	8	2
	*Hd*	0.8103	0.5194	0.7576	0.7859	0.3126	0.4073	0.8058	0.5123
	*π*	0.0026	0.001	0.0023	0.0037	0.0007	0.0007	0.0099	0.0086
	*Tajima’s D*	−1.5735 *	−1.0372	−1.7285 *	−1.4686 *	−1.1759	−0.1594	3.205	3.281
	*Fu’s Fs*	−8.3631 *	−2.8446	−12.8094 *	−6.695 *	−2.8968	−2.3031	7.8029	13.4952

Notes: MEX = Mexico; CR = Costa Rica; EP = East Pacific; WP = West Pacific; EA = East Atlantic; WA = West Atlantic; NI = Northeast Indian; WI = West Indian. Asterisks (*) indicate statistical significance (*p* < 0.05) for Tajima’s D and Fu’s Fs tests.

**Table 2:** Genetic diversity indices of *Lepidochelys olivacea* oceanic aggregations across RMUs.

Dataset	Statistics	EP	WP	EA	WA	NI	WI
474 bp	n	31	86	95	2	2	5
	S	5	22	5	0	13	0
	H	6	16	6	1	2	1
	Hd	0.5742	0.8181	0.2701	0	1	0
	π	0.0016	0.01358	0.0006	0	0.0274	0
653 bp	n	31	86	95	2	2	5
	S	7	26	6	0	14	0
	*H*	6	22	6	1	2	1
	*Hd*	0.5742	0.8254	0.2701	0	1	0
	*π*	0.002	0.0109	0.0004	0	0.0214	0

Notes: EP = East Pacific; WP = West Pacific; EA = East Atlantic; WA = West Atlantic; NI = Northeast Indian; WI = West Indian.

## Data Availability

The original contributions presented in this study are included in the article and Supplementary Materials. Sequence data are available in NCBI GenBank under accession numbers provided in Supplementary Table S3. Raw sequencing data and alignments are available from the corresponding author upon reasonable request.

## Supplementary Materials

The following supporting information can be downloaded at https://www.mdpi.com/article/10.3390/ani16193021/s1. Table S1: Haplotype nomenclature and frequencies for short-fragment sequences (474 bp); Table S2: Haplotype nomenclature and frequencies for long-fragment sequences (653 bp); Table S3: Complete metadata including GenBank accession numbers, sampling locality, RMU, aggregation type (nesting or oceanic), and sequence for all compiled samples. The GenBank accession numbers allow the original source of each sequence to be traced; Table S4: Alignment files with standardized haplotype names; File S1: Correspondence table linking previously published haplotype names to the newly proposed nomenclature (LoA for Atlantic haplotypes; LoIP for Indo-Pacific haplotypes).
