# Supplementary material for: Phylogeography of the Olive Ridley Sea Turtle (Lepidochelys olivacea): Standardized Global Haplotype Nomenclature and Updated Mitochondrial Lineage Distributions in the Eastern Pacific
Source: Animals (Basel). 2026 Sep 25;16(19):3021. doi: 10.3390/ani16193021 (PMC13663389; doi:10.3390/ani16193021)
Supplement: Supplementary file 1 [file animals-16-03021-s001.zip › Table_S4.pdf]

Supplementary Table S4

Correspondence table linking previously published haplotype names to the newly proposed standardized nomenclature for *Lepidochelys olivacea*

Table S4. Correspondence between previous haplotype assignments and the standardized nomenclature proposed in this study for *Lepidochelys olivacea*, based on two fragment lengths (474 bp and 653 bp). The table lists all previously published haplotype names, their corresponding new designations, the Haplowebmaker identifier, the geographic region of assignment, a representative GenBank accession number, and the total frequency observed in this study.

Previous Assignment	New Name (474 bp)	New Name (653 bp)	Haplowebmaker ID	Region	GenBank Accession	Total Frequency
**F**	LoA1	LoA1.1	1	Atlantic	AF051773	414
**N**	LoIP1	LoIP1.1	2	Indo-Pacific	AF051776	194
**Lo1**	LoIP2	LoIP2.1	3	Indo-Pacific	JN391445.1	113
**K**	LoIP3	LoIP3.1	4	Indo-Pacific	AF051775	48
**Unspecified**	LoIP4	LoIP4.1	6	Indo-Pacific	MK414428	27
**Lo2**	LoIP5	LoIP5.1	7	Indo-Pacific	JN391446.1	28
**K**	LoIP6	LoIP6.1	8	Indo-Pacific	AY920519.1	24
**Lo3**	LoIP7	LoIP7.1	9	Indo-Pacific	JN391447.1	22
**Lo014**	LoA2	LoA2.1	8	Atlantic	FJ795404	27
**E/Lo52**	LoIP8	LoIP8.1	10	Indo-Pacific	AY920522	21
**Lo-T1**	LoIP9	LoIP9.1	11	Indo-Pacific	KX768696	16
**M/Lo60**	LoIP10	LoIP10.1	13	Indo-Pacific	AY920520.1	16
**Lo99**	LoIP11	LoIP11.1	14	Indo-Pacific	MN342236	16

**Lo19**	LoIP12	LoIP12.1	15	Indo-Pacific	JN391463.1	14
**Lo8**	LoIP13	LoIP13.1	16	Indo-Pacific	JN391452	15
**Lo28**	LoIP14	LoIP14.1	17	Indo-Pacific	MW221480.1	12
**Lo94**	LoA4	LoA4.1	18	Atlantic	VILACA.24	12
**Lo-T6/Lo61**	LoIP15	LoIP15.1	19	Indo-Pacific	KX812522.1	11
**Unspecified**	LoA5	LoA5.1	20	Atlantic	MN047281	9
**Lo93**	LoA6	LoA6.1	21	Atlantic	VILACA.17	9
**N**	LoIP17	LoIP17.1	22	Indo-Pacific	AY920521.1	8
**Lo27**	LoIP18	LoIP18.1	23	Indo-Pacific	KC207830	8
**Lo92**	LoA7	LoA7.1	25	Atlantic	VILACA.45	8
**Lo4**	LoIP20	LoIP20.1	26	Indo-Pacific	JN391448.1	7
**J**	LoIP19	LoIP19.1	28	Indo-Pacific	AF051774	7
**Lo11**	LoIP16	LoIP16.1	29	Indo-Pacific	JN391455.1	8
**Lo-2/Lo25**	LoIP23	LoIP23.1	30	Indo-Pacific	KM357630.1	6
**Lo59**	LoIP24	LoIP24.1	31	Indo-Pacific	OP821914.1	6
**Lo027**	LoA3	LoA3.1	32	Atlantic	FJ795409	21
**Lo7**	LoIP22	LoIP22.1	33	Indo-Pacific	JN391451.1	5
**Lo12**	LoIP25	LoIP25.1	34	Indo-Pacific	JN391456	5
**Lo31**	LoIP28	LoIP28.1	35	Indo-Pacific	OP821923	5

**Lo-T7/Lo28**	LoIP26	LoIP26.1	36	Indo-Pacific	KX768697.1	5
**Lo52**	LoIP27	LoIP27.1	37	Indo-Pacific	OP821916	5
**Lo95**	LoA8	LoA8.1	38	Atlantic	VILACA.51	5
**Lo6**	LoIP21	LoIP21.1	39	Indo-Pacific	JN391450	7
**Unspecified**	LoA9	LoA9.1	40	Atlantic	MN047284	4
**Lo-5**	LoIP29	LoIP29.1	43	Indo-Pacific	KM357633	3
**Lo55**	LoIP30	LoIP30.1	44	Indo-Pacific	VILACA.3.1	3
**Unspecified**	LoIP31	LoIP31.1	47	Indo-Pacific	KY091849	2
**Unspecified**	LoIP32	LoIP32.1	48	Indo-Pacific	MK749421	2
**O**	LoIP33	LoIP33.1	49	Indo-Pacific	AY920523.1	2
**Lo21**	LoIP34	LoIP34.1	50	Indo-Pacific	JN391465.1	2
**Lo98**	LoIP35	LoIP35.1	51	Indo-Pacific	MN342237	2
**Lo5**	LoIP36	LoIP36.1	52	Indo-Pacific	MW221478	2
**Unspecified**	LoIP37	LoIP37.1	53	Indo-Pacific	MZ043567	2
**BR2**	LoA10	LoA10.1	47	Atlantic	DQ177335	1
**Lo5**	LoIP38	LoIP38.1	54	Indo-Pacific	JN391449	1
**Lo20**	LoIP39	LoIP39.1	55	Indo-Pacific	JN391464	1
**Lo-1**	LoIP40	LoIP40.1	56	Indo-Pacific	KM357629	1

**Lo-T8/Lo108**	LoIP41	LoIP41.1	57	Indo-Pacific	KX768698	1
**Unspecified**	LoIP42	LoIP42.1	58	Indo-Pacific	KY091849	1
**Unspecified**	LoIP43	LoIP43.1	59	Indo-Pacific	MH375631	1
**Unspecified**	LoIP44	LoIP44.1	60	Indo-Pacific	MK414430	1
**Lo62**	LoIP45	LoIP45.1	61	Indo-Pacific	MK749419	1
**Lo73**	LoIP46	LoIP46.1	62	Indo-Pacific	MK749420	1
**Lo96**	LoIP47	LoIP47.1	63	Indo-Pacific	MK749421	1
**Unspecified**	LoIP48	LoIP48.1	64	Indo-Pacific	MZ043568	1
**Unspecified**	LoIP49	LoIP49.1	65	Indo-Pacific	MZ043569	1
**Unspecified**	LoIP50	LoIP50.1	66	Indo-Pacific	MZ043570	1
**Unspecified**	LoIP51	LoIP51.1	67	Indo-Pacific	MZ043571	1
**Unspecified**	LoIP52	LoIP52.1	68	Indo-Pacific	OK040401	1
**Unspecified**	LoIP53	LoIP53.1	69	Indo-Pacific	OK040407	1
**Unspecified**	LoIP54	LoIP54.1	70	Indo-Pacific	OK040408	1
**Lo87**	LoIP55	LoIP55.1	71	Indo-Pacific	OP821912	1
**Lo53**	LoIP56	LoIP56.1	72	Indo-Pacific	OP821915	1
**Lo60**	LoIP57	LoIP57.1	73	Indo-Pacific	OP821918	1

**Lo62**	LoIP58	LoIP58.1	74	Indo-Pacific	OP821919	1
**Lo37**	LoIP59	LoIP59.1	75	Indo-Pacific	OP821927	1
**Lo107**	LoIP60	LoIP60.1	76	Indo-Pacific	OR400416	1
**Unspecified**	LoIP61	LoIP61.1	77	Indo-Pacific	U40657	1
**Unspecified**	LoIP62	LoIP62.1	78	Indo-Pacific	U40661	1
**Lo83**	LoIP63	LoIP63.1	79	Indo-Pacific	VILACA.116	1
**LoX**	LoIP64	LoIP64.1	80	Indo-Pacific	VILACA.123	1
**Lo98**	LoIP65	LoIP65.1	81	Indo-Pacific	VILACA.80	1

Notes

1. **Previous Assignment:** Haplotype names used in previously published studies or public databases (e.g., NCBI GenBank or Vilaça et al. [4]).
2. **New Name (474 bp):** Standardized haplotype designation for sequences aligned to the 474 bp fragment. Atlantic haplotypes are designated as LoA; Indo-Pacific haplotypes are designated as LoIP.
3. **New Name (653 bp):** Standardized haplotype designation for sequences aligned to the 653 bp fragment. Subhaplotypes resolved only in the longer fragment are indicated by ".1", ".2", etc.
4. **Haplowebmaker ID:** Unique identifier assigned to each haplotype during haplotype network construction.
5. **Region:** Geographic affiliation based on the current known distribution of each haplotype (Atlantic or Indo-Pacific). These designations represent operational groupings for standardization purposes and do not imply fixed geographic origins.
6. **GenBank Accession:** Representative GenBank accession number for each haplotype. Accession numbers beginning with "VILACA." refer to haplotypes reported in Vilaça et al. [4] that lack individual GenBank accession numbers.
7. **Total Frequency:** Total number of sequences carrying each haplotype across all compiled samples in this study (nesting and oceanic aggregations combined).
8. **Subhaplotypes:** Haplotypes that differ in the 653 bp alignment but become identical after truncation to 474 bp are grouped under the same short-fragment haplotype designation (e.g., LoA2.1, LoA2.2, and LoA2.3 all correspond to LoA2 in the 474 bp dataset).

9. **Future additions:** New haplotypes should be added by continuing the numerical sequence (e.g., LoA11, LoIP66) and, where applicable, as new subhaplotypes (e.g., LoA2.4) when they differ only in the extended 653 bp region. Researchers are encouraged to consult this table before assigning new names to avoid duplication.
